# Complicated Congenital Pulmonary Adenomatoid Malformation Type I following Right Lower Lobectomy: A Case Report

**DOI:** 10.31729/jnma.8409

**Published:** 2024-01-31

**Authors:** Preeti Basnet, Anish Joshi, Saurab Karki, Suchitra Shrestha, Sagar Thapa

**Affiliations:** 1 Department of Pediatrics, Kathmandu University School of Medical Sciences, Dhulikhel, Kavrepalanchok, Nepal; 2 Nepalese Army Institute of Health Sciences, Sanobharyang, Kathmandu, Nepal

**Keywords:** *case reports*, *neonates*, *pneumonia*

## Abstract

Congenital pulmonary airway malformation is a rare congenital abnormality of the lungs. It can manifest at any age and can lead to significant morbidity and mortality in infants and children. Some individuals with congenital lung malformations may present with respiratory symptoms right after birth, while others may remain asymptomatic for extended periods. We present a case of a 4-year- old female child who experienced recurrent chest infections. Imaging revealed type I congenital pulmonary airway malformation with an underlying infection. Despite the increased risks associated with surgery and the complexity of the disease, the patient underwent a posterolateral thoracotomy with resection of the right lower lobe. The patient achieved successful outcomes and was able to recover successfully following the surgery. This case study holds significance because several studies have focused on the timing and outcomes of surgical intervention in asymptomatic cases, there remains a lack of consensus regarding symptomatic patients and their outcomes after surgery.

## INTRODUCTION

Congenital pulmonary airway malformation (CPAM) are rare parenchymal lung abnormality that is mostly found in children with the reported incidence ranging from one in 8,000 to 35,000.^1^ Infants and neonates in respiratory distress account for the majority of instances. Management options encompass an expectant approach for asymptomatic cases, symptomatic treatment to alleviate discomfort, or considering elective surgery as a potential course of action.^2^ We report a rare case of CPAM, presenting with recurrent chest infections and, underwent a lobectomy. The case report also highlights the clinical presentation and management options for this condition.

## CASE REPORT

A 4-year-old female presented to our hospital with a 7-day history of fever and cough. The fever was intermittent, reaching a maximum recorded temperature of 103°F, partially controlled by taking medicine. It was also associated with a cough, which was dry and persisted throughout the day with no post-tussive vomiting. There was no history of fast breathing, chest indrawing, weight loss, loose stool negative history of contact with tuberculosis or COVID-19 patients. For the past week, she had been taking antibiotics prescribed by a nearby health clinic, but her symptoms did not improve completely. She also had a history of previous hospital admission three months back, where she received treatment for pneumonia with intravenous antibiotics. Although she was born through an uneventful vaginal delivery, the mother did not receive any prenatal care.

At the presentation, the patient appeared drowsy and had a body temperature of 101.3°F with stable hemodynamic status. Examinations were unremarkable except for coarse crepitations that were detected in the right mammary and inframammary regions. As for the patient's investigation, her total leucocyte count was 14800/ul with neutrophilic leukocytosis with normal haemoglobin and platelet count. GeneXpert was negative for tuberculosis. Chest radiograph revealed the presence of multiple cystic changes in the lungs.

**Figure 1:**
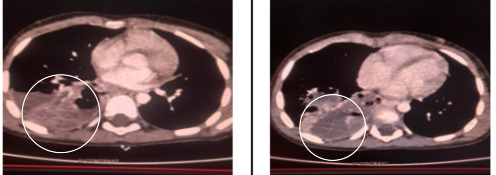
CECT of chest showing multiple variablesized fluid-filled cystic lesions in the right lower lobe with mediastinal lymphadenopathy.

The findings were further supported by a contrast- enhanced computed tomography (CECT) scan of the chest, which revealed the presence of various-sized cystic lesions filled with fluid in the right lower lobe, along with mediastinal lymphadenopathy as shown in (Figure 1).

**Figure 2:**
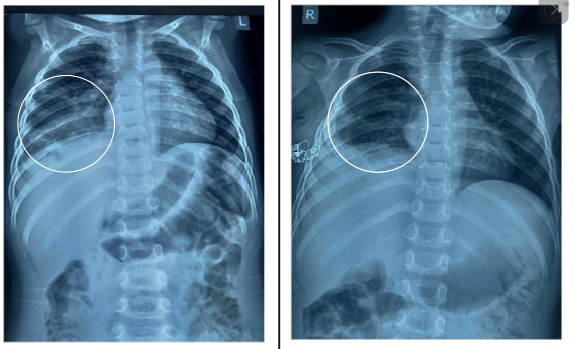
Preoperative Chest radiograph showed multiple cystic changes in the Right lung field (left side), and post-operative Chest radiograph showed the absence of cystic changes in the right lung field (right side).

These findings were suggestive of a likely type I CPAM with an underlying infection. Additionally, echocardiography was done, showing no abnormalities. The patient was initiated on intravenous antibiotics for treatment of her acute chest infection which started to resolve. Three months later, she subsequently underwent a posterolateral thoracotomy with a right lower lobe lobectomy. During the procedure, approximately 200 ml of pus was found within solid tissue, along with thickened parietal and visceral pleura. Multiple cystic changes were observed in the lung, with the largest measuring 5×9 cm, and the cysts were filled with pus. Following the procedure, she was monitored in the intensive care unit (ICU) for 11 days before being transferred to the general ward. A postoperative chest radiograph did not show any visible cystic changes (right side) as compared to a preoperative X-ray (left side) as shown in (Figure 2).

The patient was discharged on oral antibiotics along with medicines for symptomatic care. She was clinically stable on her follow-up visit and showed no signs of complications. The histopathology examination report indicated findings suggestive of bronchopulmonary pneumonia in the background of CPAM.

## DISCUSSION

In 1949, Chin and Tang first identified congenital pulmonary adenomatoid malformation (CPAM) as a distinct condition. The underlying cause of CPAM is generally attributed to pulmonary maldevelopment in which a portion of the lung or an entire lobe is replaced by non-functional tissue, which may be aplastic or consist of micro- or macro cystic pulmonary tissue.^3^ The most common time for CPAM to be detected is during the standard 18-20 week scan, where the prenatal diagnosis rate for lung cysts is nearly 100% however, the sensitivity of diagnosis decreases during late pregnancy.^4^ In this case, the condition was not detected antenatally due to irregular antenatal care (ANC) obstetric scans conducted during the first and second trimesters.

Although the clinical signs and symptoms can vary, the primary clinical finding is recurrent lung infections.^5^ While a chest radiograph has a limited sensitivity of approximately 60% in identifying CPAM, a CT scan is considered the most effective imaging tool for postnatal diagnosis.^6^ In this case, the patient experienced recurrent chest infections after the first year of life, the previous chest radiograph showed no abnormalities however, the cystic lesion associated with CPAM was only detected much later. Performing elective surgery for CPAM yields better outcomes, particularly when performed on asymptomatic newborns, as it reduces the risk of complications. Although the optimal timing for cyst removal is uncertain, it should be conducted within the first 10 months of the infant's life if surgery is deemed necessary.^7^ Opting for surgery when individuals first start experiencing symptoms of CPAM carries certain risks. These include a longer hospital stay, extended pleural drainage, and the need for invasive ventilation. Additionally, there is a higher incidence of postoperative complications such as fistula, haemorrhage, and the potential need for a second surgery. However, it is important to note that early surgery allows for better compensation as the lung continues to develop and grow until the age of four. The growing lung has the potential for improved adaptation and compensatory mechanisms, which can contribute to better outcomes if surgery is performed at an earlier stage.^8^

Therefore, CPAM being a rare condition, can be detected during ANC visits, highlighting the importance of early scans. The complex presentation of CPAM where the patient had persistent and recurrent infections made the treatment challenging however, she experienced successful outcomes and was able to recover completely after the surgery. CPAM can present with a range of symptoms. In children presenting with recurrent chest infections and respiratory distress, it can be one of the possible differential diagnoses. Performing elective surgery early in life can yield better outcomes in children.
